# A Pleistocene Clone of Palmer's Oak Persisting in Southern California

**DOI:** 10.1371/journal.pone.0008346

**Published:** 2009-12-23

**Authors:** Michael R. May, Mitchell C. Provance, Andrew C. Sanders, Norman C. Ellstrand, Jeffrey Ross-Ibarra

**Affiliations:** 1 Section of Ecology and Evolution, University of California Davis, Davis, California, United States of America; 2 Department of Botany and Plant Sciences, University of California Riverside, Riverside, California, United States of America; 3 Department of Botany and Plant Sciences and Center for Conservation Biology, University of California Riverside, Riverside, California, United States of America; 4 Department of Plant Sciences, University of California Davis, Davis, California, United States of America; University of Melbourne, Australia

## Abstract

**Background:**

The distribution of Palmer's oak (*Quercus palmeri* Engelm.) includes numerous isolated populations that are presumably relicts of a formerly larger range that has contracted due to spreading aridity following the end of the Pleistocene.

**Principal Findings:**

We investigated a recently discovered disjunct population of Palmer's oak in the Jurupa Mountains of Riverside County, California. Patterns of allozyme polymorphism, morphological homogeneity, widespread fruit abortion, and evidence of fire resprouting all strongly support the hypothesis that the population is a single clone. The size of the clone and estimates of annual growth from multiple populations lead us to conclude that the clone is in excess of 13,000 years old.

**Conclusions:**

The ancient age of the clone implies it originated during the Pleistocene and is a relict of a vanished vegetation community. Range contraction after climate change best explains the modern disjunct distribution of *Q. palmeri* and perhaps other plants in California.

## Introduction

**Figure 1:**
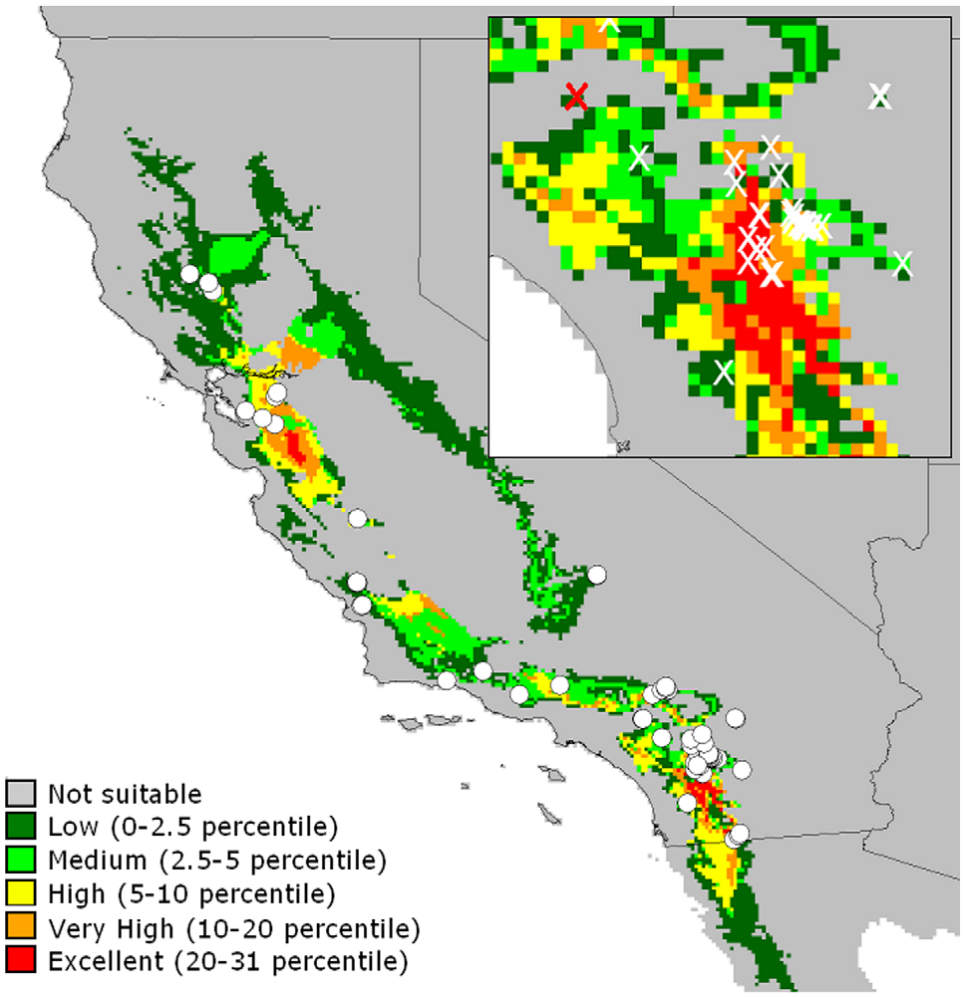
Map of the studied population of *Q. palmeri*. White dots and ‘X’s represent known occurrences of *Q. palmeri* throughout California; the red ‘X’ in the insert represents the location of the Jurupa population. Percentiles are the two-tailed probability of suitability given 19 bioclimatic parameters.

Numerous woody species of plants in the southwestern United States exhibit scattered distributions characterized by small, isolated populations, often occurring in suboptimal habitats [1]. One explanation for these scattered distributions is range contraction resulting from spreading aridity and increased temperatures following the end of the last glacial period in the late Pleistocene [2]. One species that demonstrates this scattered distribution is Palmer's Oak (*Quercus palmeri* Engelm., = *Q. dunnii* Kellogg). Palmer's Oak is a xerophytic evergreen shrub or small tree of *Quercus* section Protobalanus. *Q. palmeri* exhibits a disjunct distribution, with populations in Arizona and California completely separated by the Mojave and Sonoran deserts. In California, populations of *Q. palmeri* are extensive in the eastern Peninsular Range of Riverside and San Diego counties, but elsewhere are typically small and separated from neighboring populations by many kilometers (Fig. 1). The species ranges from northern Baja California to north of the San Francisco Bay region. Known occurrences in California are usually between 900 and 1500 m in elevation on the desert slopes of the Transverse and Peninsular Ranges, with scattered outposts in the South Coast Ranges and southeastern Sierra Nevada Mountains. Populations are typically associated with mesic sites in the arid interior, though many occur at the margins of springs and on deep soils in montane valleys.

An isolated occurrence of *Q. palmeri* was recently discovered in the Jurupa Mountains of Southern California (34.033°N, 117.391°W, elevation 366 m), in drier habitat and at a lower altitude than previously reported for this species [3]. The bulk of the stems at the Jurupa site are in a narrow gulch between two large granite boulders on a relatively steep north-facing slope at an elevation of about 400 m, in a coastal sage scrub community. There is no other record of *Q. palmeri* in the Jurupa area, and local floras do not report this species from any of the surrounding areas, except for a recent discovery in the Bernasconi Hills west of the San Jacinto River [4]. At the Jurupa site, *Q. palmeri* is represented by approximately 70 stem clusters forming a dense and homogeneous thicket with dimensions of approximately 25×8 m and limited to roughly 1 m in height by some combination of strong northerly winds, drought, and fire [3]. Patterns of stem emergence and high stem density make it difficult to determine the number of distinct individuals at the site by observation alone.

When first observed, morphological homogeneity, high rates of acorn abortion, and abundant evidence of resprouting following fire suggested that all stems of *Q. palmeri* at this location might belong to a single clone. Evidence for clonal propagation has been suspected in other occurrences of *Q. palmeri* [5], and it is possible that many of the smaller, isolated populations of *Q. palmeri* are in fact single clones [6]. We present allozyme data confirming the clonal nature of the stand at Jurupa, as well as indirect estimates of the age of the clone that suggest its persistence since the Pleistocene.

## Methods

Individual stems in the Jurupa population were tagged and numbered. Vouchers of the population were deposited in the UCR Herbarium. Plants were examined for maturing fruits and new growth eight times over a six year period. Comparative specimens were collected from larger, actively reproducing, Californian populations of *Q. palmeri* in Garner Valley (33.575°N, 116.6°W, elevation 1440 m) and a location near Aguanga (33.483°N, 116.75°W, elevation 1265 m). Evidence of root sprouting or rhizome formation in the Jurupa stand was sought by excavation of the soil at the base of several stems.

### Allozyme Electrophoresis

Newly mature leaf tissue was collected from across the Jurupa site, including 32 of the roughly 70 stem clusters, ensuring inclusion of all potential clonal groupings. Leaf tissue was also sampled from fifteen well-spaced trees and shrubs among the thousands of *Q. palmeri* present in the Garner Valley population. Collections were immediately taken to the laboratory for protein extraction.

Protein was extracted from leaf samples using liquid nitrogen and a modified version of the extraction buffer of Mitton *et al.* [7]. The resulting extract was then absorbed onto paper wicks, which were stored at −80°C for later use. Starch gel protein electrophoresis using three different buffer systems resolved a total of 11 loci. A modified morpholine-citrate buffer system [8] resolved 4 loci: leucine aminopeptidase (LAP), malate dehydrogenase (MDH), Shikimate dehydrogenase (SKDH), and fluorescent esterase (FE). A discontinuous lithium hydroxide-borate buffer system [9] resolved 4 loci: menedione reductase (MNR), two phosphoglucoisomerase (PGI) loci, and triose phosphate isomerase (TPI). Finally, a pH 6 histidine-citrate buffer system [10] resolved 3 loci: two phosphoglucomutase (PGM) loci, and uridine-5′-diphosphatase (UDP). Both PGM loci were dropped from the analysis due to poor quality bands and inconsistent banding patterns among replicates. Specimens of the related Golden Oak (*Q. chrysolepis* Liebm.) were analyzed alongside *Q. palmeri* to confirm the similarity of banding patterns to those reported by Montalvo *et al.* [11].

### Age Estimation

**Figure 2:**
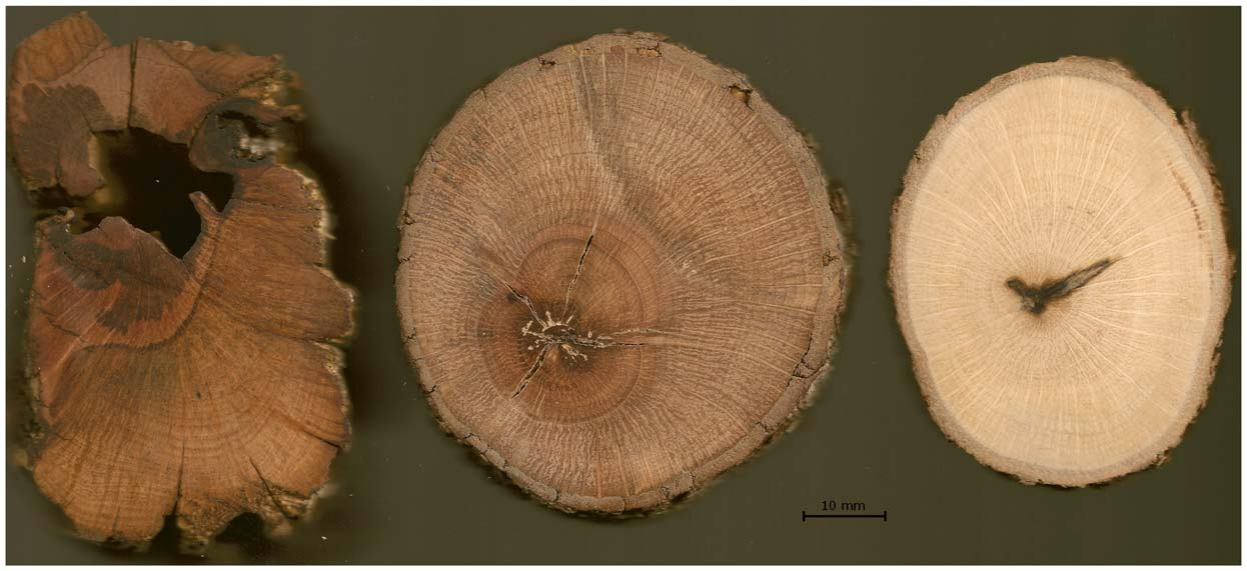
Cross-sections of *Q. palmeri* stems. Ring counts were made from high-resolution images of cross sections of each stem. Scale is in millimeters. Example stem sections, from left to right: Jurupa Mountains, Aguanga, Garner Valley.

Excavations at the Jurupa site failed to produce any significant amounts of old, dead wood. This absence of old material, likely due to termite activity, made it impossible to determine the age of the putative clone directly by means of radiocarbon dating. Instead, we counted annual growth rings in multiple stem cross-sections to determine average annual growth rate. Transverse sections of 10 dead stems and 1 live branch were collected from different locations within the Jurupa site. Cross-sections were air-dried, finely sanded, and in some cases stained to make annual growth rings visible. Ring counts and diameters were estimated manually from digital images of the sections (Fig. 2, additional images available at http://www.rilab.org/treering/treering.html). Average annual growth rate was determined from the number of annual growth rings present over a given distance in individual cross-sections. Growth rates were similarly estimated from stems collected at two additional sites in Garner Valley and near Aguanga.

### Environmental Suitability

We gathered occurrence data for *Q. palmeri* from herbarium databases available at Calflora (http://www.calflora.org/) and the Consortium of California Herbaria (http://ucjeps.berkeley.edu/consortium/). We used these occurrence data to perform ecological niche modeling in the software package DIVA-GIS [12], using data on 19 bioclimatic variables drawn from Worldclim [13] and elevation data from The Shuttle Radar Topography Mission [14].

## Results

Genotypic data from nine allozyme loci for approximately half of the stems at the Jurupa site strongly support the conclusion that the stand is a single clone. All nine loci showed identical banding patterns in every stem analyzed. Most loci revealed single-band patterns consistent with homozygous genotypes, but two of the loci (FE and PGI) revealed a two-band pattern suggestive of fixed heterozygosity. Comparisons to variation observed in individuals from the Garner Valley population and samples of *Q. chrysolepis* reinforce these data, as each of the homozygous loci in the Jurupa population was observed to be polymorphic in other samples, and both of the fixed heterozygous loci were seen to segregate in a normal Mendelian fashion (data not shown). While high levels of homozygosity may simply represent elevated inbreeding (as demonstrated by a study of Wollemi pine, *Wollemia nobilis* [15]), the observation of fixed heterozygous genotypes is extremely unlikely except in a clonal population. Even under the best circumstances (when allele frequencies are equal and mating is random), the probability that 32 individuals would be heterozygous at a given locus is vanishingly small (<3×10^−10^). There is thus little chance the various stems at Jurupa represent genetically different individuals.

Morphological homogeneity of the Jurupa stand supports the genetic data. Overall growth form and leaf morphology is strikingly uniform throughout the site, and the vast majority of stems appeared relatively healthy. Stem clusters closely resemble those reported from other small, isolated populations of *Q. palmeri* from northern parts of California [5, 16, Sanders, personal observation]. All stems in the Jurupa site were in flower when discovered. Despite prolific flowering, however, we found virtually no evidence of sexual reproduction: tiny, aborted acorns were abundant in the leaf litter, and over the course of many visits to the site spanning more than six years we observed no seedlings and only four mature acorns. Attempts to germinate acorns from the Jurupa site in a greenhouse failed, whereas acorns collected from the Garner Valley population demonstrated normal viability under the same conditions (data not shown). We also noted significant signs of recent fire damage at the Jurupa site, including fire-killed stems up to 1 m tall. Several scorched trunks were evident in the center of the largest clump of individuals, and smaller pieces of carbonized material were found throughout the site. No evidence of adventitious shoot production from roots or of production of rhizomes was observed in the Jurupa population. New shoots following fire appear to be essentially vertical and grow from existing buds from the root crown at the base of burned stems.

If *Q. palmeri* at the Jurupa site represents a single asexually reproducing clone expanding only by secondary growth in stem thickness due to crown resprouting after fire, comparison of the diameter of the clone to measurements of annual growth provides a means of estimating the age of the clone. Making the conservative assumption that the progenitor individual originated at the center of the current Jurupa site, the clone must have grown at least 12.5 m (half the longest axis) in a single direction. We estimated the age of the clone under two different growth rate scenarios: 1) average growth rate observed using stems at the Jurupa site, taken to represent the growth rate of *Q. palmeri* in poor conditions; and 2) average growth rate observed across all three populations sampled, assumed to represent a wide range of growth conditions. If only stems from the Jurupa site are included, we estimate an average annual growth rate of 0.8±0.02 mm per year, resulting in an age range of approximately 15,600±2,500 years. The average annual growth rate determined from all stems collected from all populations was 0.96 mm per year, providing a minimum age estimate of approximately 13,000 years–this is our most realistic estimate of the age of the clone, since it takes into account a wide range of growth conditions.

**Figure 3:**
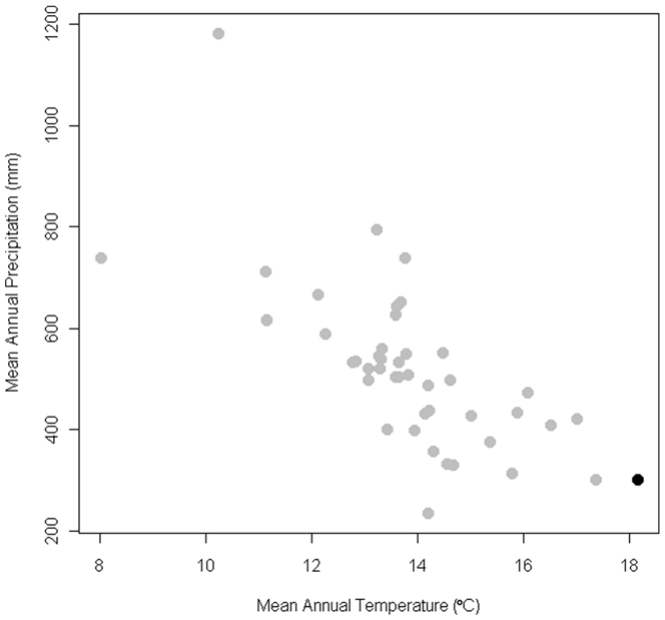
Comparison of mean annual temperature and mean annual precipitation among occurrences of *Q. palmeri*. The black dot represents the *Q. palmeri* at the Jurupa Mountains site. The Jurupa site is outside the one-tailed 95% confidence interval for both variables.

Ecological niche modeling of altitudinal and bioclimatic data linked to 44 known occurrences of *Q. palmeri* in California demonstrates that the Jurupa Mountains site is suboptimal for growth of *Q. palmeri* (Fig. 1). Most notably, the Jurupa site has the highest mean annual temperature and the second-lowest mean annual precipitation of all of the sampled occurrences (Fig. 3).

## Discussion

Data from allozyme analysis, combined with morphological and phenological observation, provide clear evidence that the Jurupa population of *Q. palmeri* is a single clone. Samples taken from all of the spatially distinct clumps of individuals, including nearly half of the stem clusters in the stand, show no genotypic variation at 9 allozyme loci, including fixed heterozygosity at two loci. The stand exhibited morphological uniformity and a multi-stem, clumped growth form, facts that Tucker *et al.* associate with clonality in *Q. palmeri* populations in northern California [5]. This inference is reinforced by the frequency of acorn abortion and the extreme rarity of mature acorns found in the population: oaks, like most predominantly outcrossing plants, are expected to suffer loss of productivity when clone density is high [17]. In marked contrast to the frequent fruit abortion in the Jurupa population, an extensive population in Garner Valley produces large numbers of viable acorns. Several other populations of *Q. palmeri* exhibit characteristics suggestive of clonal growth [5], and it has been suggested that many of the smaller, disjunct populations are single clones [6]. The data presented here are the first, however, to substantiate clonal growth in *Q. palmeri*.

We measured average annual growth rates in stems of the Jurupa clone and in stems of some of the most favorable sites known to us, with the idea of estimating clonal age by comparing annual growth to the size of the clone. The large populations of *Q. palmeri* in Garner Valley and near Aguanga doubtless represent populations growing under something close to optimal conditions for the species. We assume that the current average growth rate at the Jurupa site, where only a single clone survives at an extraordinarily low and dry site for the species, must represent something close to the slowest growth rates normally exhibited by the species. Our most realistic estimate, based on stems collected from a variety of environmental conditions, is that the clone is at least 13,000 years old.

Sources of error in our estimates of the age of the Jurupa clone fall broadly into two categories: the mode and rate of clonal growth, and the size of the clone. Other forms of clonal growth, such as root sprouting or rhizome formation, could result in higher rates of clone diameter growth, making our estimate of the average annual increment too low and our estimate of the time required for the clone to spread to its present dimensions too great. Other oak species are known to spread rhizomatously [18] or by root sprouting [19]; the well-studied *Q. chrysolepis*, a close relative of *Q. palmeri*, is known to spread by root sprouting [20], but regrowth after fires has only been observed via crown resprouting [21], [22], and extensive observation of clones of this species show no evidence for any other form of growth [11]. Moreover, our excavations at the Jurupa site offered no evidence for either rhizomatous expansion or root sprouting. Our estimation also assumes that crown resprouts occur in all directions after a fire, such that the clonal axis is representative of the path of clonal spread. If crown resprouting is inconsistent, however, the spread of the clone need not be linear and our age calculations would be underestimates. Anomalous growth rates could also have the effect of biasing our estimates in either direction: poor growth conditions, for example, could result in narrow annual growth rings, leading to an overestimation of the age, but could also result in a complete lack of growth in some years, biasing our estimate downward.

In addition to variability in growth form, the exact size of the clone and the position of the original ramet could also influence age estimation. Noticeable fragmentation and considerable dead wood at the top of the ridge suggests the Jurupa clone may once have extended over the ridge top or elsewhere out of the relatively well-protected gully to which it is now restricted. Perhaps the most significant source of error in our estimations comes from our assumption that the progenitor individual originated in the center of the current clone. If the position of this original ramet were anywhere else–potentially even outside the current stand–our estimates would be considerably lower than the clone's true age. In fact, both competitive and environmental effects have been shown to act to limit clone size in other tree species [23], and these limitations likely result in underestimation of clone age [24]. Because of these many sources of error–particularly the unknown position of the original ramet and the effects of ecological limitations on stand spread, which result in potentially significant and unknowable underestimates of clone age–we stress that our estimates of age are not absolute, but represent a minimum age for the Jurupa clone.

Our age estimates place the original germination of the Jurupa stand of *Q. palmeri* and the beginning of clonal spread in the late Pleistocene. There is no climatological evidence to suggest that conditions at the glacial maximum were such that *Q. palmeri* could not have grown in the Jurupa Mountains during the late Pleistocene. The maximum extent of local glaciation during the last 30,000 years was on the upper slopes of San Gorgonio Mountain [25], 90 km northeast and 2,000 m higher in altitude than the Jurupa site. In fact, the period from 25,000–40,000 years ago supported a diverse woodland flora at Rancho La Brea, about 100 km to the west, including *Cupressus, Pinus*, *Sequoia, Juglans* and *Quercus* [26], suggesting that conditions were then more favorable for growth of *Q. palmeri* at a locality that is today quite similar in climate to the Jurupa Mountains. Pollen records from packrat middens show that *Q. palmeri* occurred in the Mojave Desert at an elevation of 850 m starting 9,500 years ago, replacing the previously dominant *Pinus monophylla* [27], which today occurs at a mean altitude higher than *Q. palmeri*. The mean elevation of occurrence of other woody plant species in Southern California, including *Q. chrysolepis*, has been shown to increase in response to climatic warming [2]. It is thus reasonable to assume that *Q. palmeri* existed at lower elevations in the past than it does today. The fact that the Jurupa clone currently exists at a lower elevation in a drier, hotter habitat than the rest of the species is therefore consistent with our estimated age.

We propose that this stand of *Q. palmeri* is a relict of an ancient population that has persisted in the Jurupa Mountains despite warming since the last glacial period. Our findings are not without precedent, as ancient clones have been identified in other woody taxa [28]–[30], including a nearly 12,000 year old clone of creosote (*Larrea tridentata* Coville) found in the Mojave Desert [31]. Nonetheless, our 13,000 year estimate for the age of the Jurupa clone places it among the oldest of living plants.

Finally, we have proposed that warming since the last glacial period has pushed the ideal elevation of *Q. palmeri* higher, leaving behind the small, disjunct populations scattered around California today. Our findings at Jurupa suggest that cloning may be a significant contributor to the persistence of these disjunct populations. Numerous other woody shrubs in the southwest share such disjunct distributions and patterns of growth [1], and it is thus tempting to speculate that disjunct populations of many of these other species may consist of extremely long-lived clones as well.
